# Case Report: Lifestyle changes and probiotic supplementation for improving longstanding type 2 diabetes in a male undergoing testosterone replacement therapy

**DOI:** 10.3389/fendo.2025.1754430

**Published:** 2026-01-12

**Authors:** Anna Griffith, Adam Perlman, Tara Karr, Marin Elise Thompson, Orville Kolterman

**Affiliations:** 1 Victory Men’s Health, O’Fallon, IL, United States; 2 Pendulum Therapeutics Inc., San Francisco, CA, United States

**Keywords:** *Akkermansia*, lifestyle intervention diabetes, probiotic, testosterone replacement, type 2 diabetes case report

## Abstract

A 52-year-old Caucasian male sought treatment for age-related testosterone decline. Past medical history included diagnosed hypertension and type 2 diabetes mellitus, with suboptimal glycemic control and no current antidiabetic therapy. He qualified for testosterone replacement therapy and agreed to address his metabolic dysfunction through enhanced lifestyle interventions and a targeted 5-strain probiotic as well as other supplements. At 3 months, the patient demonstrated improved glycemic control (hemoglobin A1c decreased from 7.2% to 5.9%) and improvement in newly documented mixed hyperlipidemia. At 6 months, testosterone levels normalized with replacement therapy, and the patient achieved modest weight reduction with favorable body composition changes: 13.2-lb decrease in fat mass, as well as a 6.1% reduction in body fat percentage and a 5.1-lb increase in skeletal muscle mass. This case demonstrates the feasibility and clinical efficacy of combining lifestyle interventions with targeted supplementation in a patient with metabolic syndrome who preferred non-pharmacological management strategies.

## 1 Introduction

Successful management of type 2 diabetes (T2D) requires integration of self-management education, medical nutrition therapy, adequate physical activity, healthy sleep habits, appropriate behavioral counseling, and, in most cases, pharmacological intervention. Patient interest and motivation are key to the success of this multifaceted approach, yet patients often harbor negative perceptions of standard care and do not maintain the necessary behavioral changes (1). Furthermore, implementation of a successful T2D management plan is often thwarted by a desire for avoidance of pharmacological therapy, which can leave the individual exposed to the deleterious effects of hyperglycemia and related potential psychological stress, further worsening metabolic control. New, highly effective pharmacological options for addressing T2D are now available, but their use is associated with side effects that impact compliance and create safety concerns. GLP-1 agonists, across multiple studies, are associated with significant rates of nausea (15 to 50%), diarrhea (10 to 30%), and vomiting (5 to 20%). Gallbladder disease, including cholelithiasis, occurs in 1 to 3% of recipients. While pancreatitis occurs in around 1% of treated patients, a direct causal relationship remains controversial (2). Use of SGLT-2 inhibitors is associated with increased incidence of fungal genital infections (2 to 6%), urinary tract infections (0.6 to 6%) and rare instances (<1%) of normoglycemic ketoacidosis and rapidly progressing, gangrenous perineal infections which may be life threatening (3). These factors have reinforced reluctance among some patients and healthcare providers to employ these potent therapies. Therefore, non-pharmacological interventions capable of both improving metabolic parameters and potentiating lifestyle modifications represent an important clinical need. This case report details the successful use of such a non-pharmacological intervention, resulting in measurable clinical benefits.

## 2 Case description

We report a case of a 52-year-old married father with a longstanding history of metabolic syndrome. The patient presented to a new provider with chief complaints of progressive decreased libido associated with poor quality sleep. He reported no difficulty falling asleep but experienced frequent awakenings and difficulty falling back asleep. The patient was not evaluated for sleep apnea but was known to snore. Based on discussion with friends, he became concerned that these symptoms could be the consequence of decreased testosterone associated with aging.

The patient’s medical history included increased waist circumference, hypertension, hyperglycemia, and recurrent urinary tract infections in childhood due to the congenital presence of an accessory ureter, which was resolved surgically at age 7. T2D was diagnosed at age 40 and initially treated with metformin and weight loss. After these interventions restored normoglycemia, metformin was discontinued. The patient lost motivation for continued lifestyle changes and his weight gradually increased over the following years, accompanied by a return of hyperglycemia that was not adequately addressed. His family history was negative for T2D. Current medications and supplements were limited to amlodipine (5mg/day) for hypertension. The patient denied use of tobacco, alcohol, or recreational drugs.

Physical examination revealed a weight of 201.7 lbs, a height of 5 feet 10 inches, BMI of 28.9, blood pressure of 136/82 mmHg, and abdominal obesity (see **Table 1**). No abnormalities were reported in external genitalia. ADAM questionnaire responses (4) were consistent with low testosterone. SHIM questionnaire score of 24 did not suggest erectile dysfunction. Relevant laboratory tests (summarized in **Table 1**) revealed total and free testosterone levels that were at the lower limits of the assay normal range, accompanied by mid-range LH, FSH, and prolactin levels. Inadequate glycemic control was confirmed with an A1c value of 7.2%. Non-fasting lipid measurements revealed elevated total cholesterol, LDL cholesterol, and triglycerides, accompanied by low HDL cholesterol. Body composition analysis using bioelectrical impedance (InBody USA, Cerritos, CA) revealed a skeletal muscle mass of 87.3 lbs and body fat mass of 48.9 lbs, corresponding to 28.3% body fat.

**Table 1:** Summary of patient data at baseline, 3 months, and 6 months.

Parameter	Baseline	3 months	6 months	Reference range
Plasma Glucose (mg/dL)	150*	113*	N/A	74 - 126
Hemoglobin A1c (%)	7.2	5.9	N/A	4 - 5.7
Triglycerides (mg/dL)	667	113	N/A	<150
Total Cholesterol (mg/dL)	213.5	177.8	N/A	0 - 200
LDL Cholesterol, Calculated (mg/dL)	N/A	121	N/A	<100
LDL Cholesterol, Direct (mg/dL)	143	N/A	N/A	<100
HDL Cholesterol (mg/dL)	38.6	34.5	N/A	>40
Vitamin D, 25-Hydroxy (ng/mL)	18.7	42.9	47.4	30 - 100
Testosterone, Total (ng/dL)	232	479	845	240 - 950
Free Testosterone, Calculated* (ng/dL)	4.99	13.4	22.6	4.06 - 15.6
Prostate Specific Antigen (PSA) (ng/mL)	0.45	0.56	0.53	0 - 4
Estradiol, sensitive (pg/mL)	<15.00	20	34.9	10 - 40
FSH (mIU/mL)	6.57	<0.20	.34	1 - 18
LH (mIU/mL)	4.31	<0.20	<0.20	1.24 - 8.62
Prolactin (ng/mL)	8.72	7.32	N/A	2.64 - 13.13
Hematocrit (%)	46.9	53.8	56	39.8 - 52
Hemoglobin (g/dL)	16.3	18	18.8	13.6 - 18
Body composition data (InBody body composition analysis)				
Date	5/9/24	7/31/24	10/30/24	
Weight (lb)	201.7	197.5	196.2	
BMI	28.9	28.3	28.2	
Body Fat Mass (lb)	48.9	42.1	35.7	
Percent Body Fat (%)	24.3	21.3	18.2	
Visceral Fat Score	10	9	7	
Skeletal Muscle Mass (lb)	87.3	88.6	92.4	

*Vermeulen formula.

Discussion with the patient led to agreement to use testosterone cream (200 mg/mL) provided by a local compounding pharmacy, 100 mg/day applied to the scrotum each morning (5). During the clinical consultation, the critical benefits of improving glucose control were discussed. The patient continued to decline the use of pharmacological options for his T2D but did express a willingness to pursue a non-drug intervention in conjunction with renewed attention to diet and exercise. This interest led to a discussion of a 5-strain probiotic (Pendulum Glucose Control, formulation WBF-038), specifically formulated to assist with the nutritional management of T2D. The probiotic contained 5 commensal bacteria from species frequently found in the digestive tract of healthy humans: *Clostridium beijerinckii*, *Clostridium butyricum*, *Akkermansia muciniphila*, *Anaerobutyricum hallii*, and *Bifidobacterium infantis*, along with the prebiotic chicory inulin.

The patient agreed to a trial of the probiotic at the recommended dose of 2 billion active fluorescent units (AFU) per day combined with renewed targets for diet and exercise, aiming for increased fiber intake plus 1g protein/lb body weight while limiting intake of simple sugars and highly processed foods. Thirty minutes of exercise at least 5 days/week, including both aerobic and resistance training, were also recommended. Omega-3 supplementation (1.3 g/day) was added along with Vitamin D3 (5000 IU/day) to address the low Vitamin D, 25 OH level. A sublingual Vitamin B12 and folate supplement was also added.

**Figure 1:**
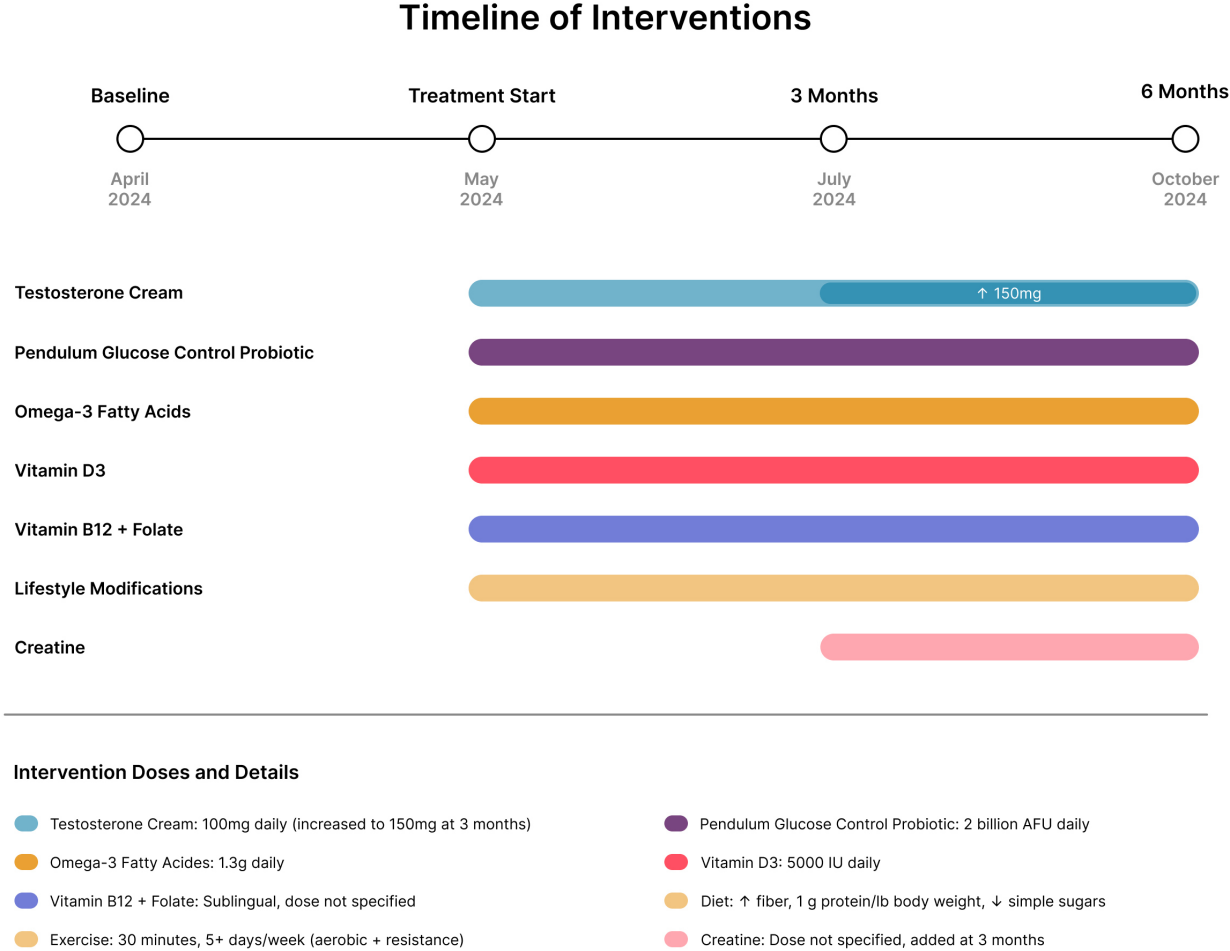
Timeline of interventions in a 52-year-old male with type 2 diabetes and age-related testosterone decline. Most interventions were initiated simultaneously at treatment start (May 2024): testosterone cream (100 mg daily, increased to 150 mg at 3 months), Pendulum Glucose Control probiotic (2 billion AFU daily), omega-3 fatty acids (1.3 g daily), vitamin D_3_ (5000 IU daily), vitamin B_12_ plus folate, and comprehensive lifestyle modifications (increased dietary fiber, protein optimization at 1 g/lb body weight, reduced simple sugars, and exercise for 30 minutes 5 or more days per week including aerobic and resistance training). Creatine supplementation was recommended and added at the 3-month follow-up visit.

For the timeline of interventions, see **Figure 1**.

## 3 Results

At his 3-month follow-up visit, the patient reported an improved libido without any noticeable changes in sleep pattern. No side effects were reported. Total testosterone level had increased to the middle of the normal range. Simultaneously, improvements in metabolic health were evident, consisting of a 4-lb weight loss, a significant reduction in A1c from 7.2% to 5.9%, and improvement in lipid parameters, as shown in **Table 1**. Blood pressure was unchanged. Body fat mass had decreased by 6.2 lb (3% decrease in body fat) and skeletal muscle mass increased by 1.3 lb. Hematocrit and hemoglobin were higher, consistent with the increased erythrocytosis associated with testosterone replacement. The improvements in metabolic health were attributed to use of the probiotic in conjunction with improved dietary practices (increased fiber intake accompanied by a decreased intake of simple sugars and highly processed foods) and increased exercise. The patient was advised to increase the dose of testosterone cream to 150 mg/day and continue on the probiotic and other supplements while maintaining the dietary and exercise regimens he had implemented. The addition of creatine supplementation was also recommended.

At the 6-month follow-up, the patient reported continued compliance with the recommended regimen, although creatine use was sporadic. The patient reported no noticeable sleep improvements. While metabolic parameters were not assessed at the visit, both testosterone and estradiol levels were near the upper limits of the normal lab range. His vitamin D level had increased to mid-range, and his body weight had decreased by 5.5 lbs compared to baseline. Body composition data, compared to baseline, revealed a decrease in body fat mass of 13.2 lbs, a 6.1% decrease in percent fat, and a 5.1-lb increase in skeletal muscle mass.

## 4 Discussion

In the present case, low libido and poor sleep quality were the primary motivators for seeking medical assistance, with an assumption that testosterone replacement therapy (TRT) would be beneficial. The patient was not initially motivated to undertake treatment for longstanding type 2 diabetes, consistent with his perception that lifestyle changes had limited effectiveness and pharmacological interventions were not of interest. A1c at presentation was 7.2% and the patient agreed to address hyperglycemia through a combination of focused lifestyle changes (increased dietary fiber and protein intake, limitation of simple sugars and highly processed foods, and regular exercise) and the addition of a probiotic specifically formulated for use in the nutritional management of type 2 diabetes. Omega-3, Vitamin D3, and Vitamin B12 and folate supplements were also added.

Follow-up visits at 3 and 6 months documented the durable success of these interventions beyond what is typically seen with lifestyle interventions alone. Use of the testosterone cream increased both total and free testosterone levels, along with estradiol levels. Vitamin D supplementation led to increased 25-OH vitamin D levels. Reinforcement of recommended dietary and exercise practices, with the daily probiotic added to nutritional T2D management, led to a significant improvement in glycemic control as evidenced by a reduction in A1c to 5.9%, a value consistent with prediabetes. These biochemical changes were associated with improved libido, reduced body fat, and increased skeletal muscle mass. The expected increase in hematocrit with TRT was apparent, with both the hematocrit and hemoglobin values being slightly above the upper limits of normal at the 6-month visit. While glucose and lipid parameters were not assessed at the 6-month visit, it is reasonable to assume that the beneficial changes seen at 3 months persisted, given maintenance of the weight loss with a further decrease in fat mass and percent body fat, accompanied by a further increase in skeletal muscle mass.

**Figure 2:**
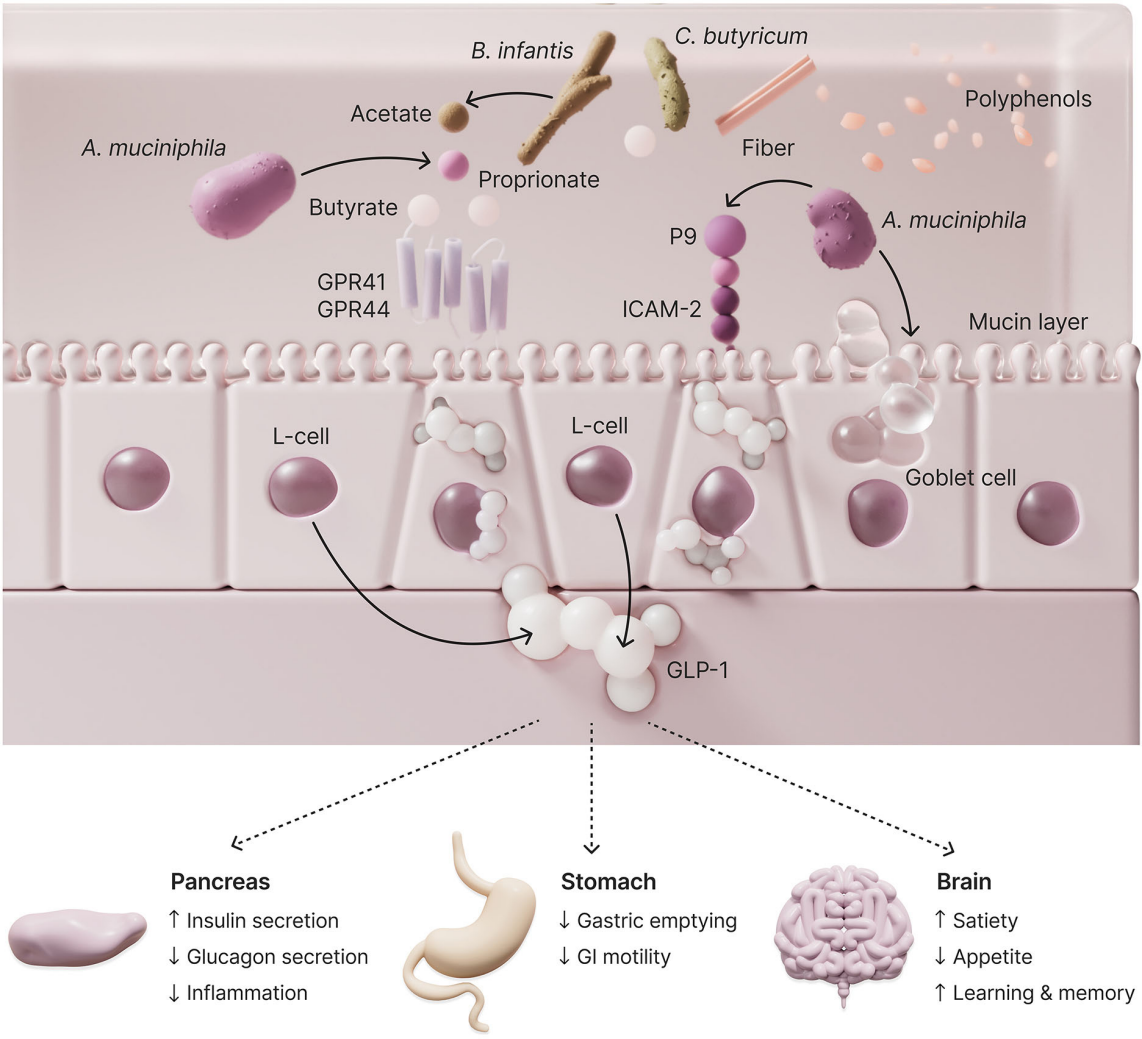
Key components of the hypothesized mechanism of action of the 5-strain probiotic for improving health in T2D. *Akkermansia muciniphila* resides within the mucin layer that overlies the intestinal epithelium, and assists in maintaining the integrity of the gut barrier. It produces propionate, a key nutrient for other microbial species. In addition, a protein constituent of *Akkermansia muciniphila* called P9 increases IL-6 to augment signaling through the L-cell ICAM-2 receptor, thereby increasing GLP-1 secretion. *Clostridium btyricum* produces butyrate through digestion of dietary fiber, and *Bifidobacterium infantis* produces acetate, cross-feeding bacteria that produce butyrate. Butyrate modulates the immune system via regulatory T cells and is also an endogenous agonist of G protein-coupled receptors 41 and 43 on L-cells, which further stimulate the secretion of GLP-1. The incretin hormone GLP-1 is important for the maintenance of glycemic control through augmentation of glucose-dependent insulin secretion, restraint of the gastric emptying rate following meal ingestion (reducing postprandial hyperglycemia), and brain effects to increase satiety and reduce food intake. glucagon-like peptide (GLP), G protein-coupled receptor (GPR), intercellular adhesion molecule (ICAM).

Many patients who discontinue lifestyle interventions do not attempt to resume them, and health professionals often do not have adequate resources to support continued implementation (6). The subject of this report had been aware of having T2D for years and having suboptimal control over it. Following a brief previous period of lifestyle measures and metformin use, he was unmotivated to pursue lifestyle interventions alone and reluctant to initiate additional pharmaceutical intervention. However, he agreed to initiate diet and exercise measures, alongside a supplement regimen that included a daily probiotic designed to enhance dietary management of T2D. While the independent contribution of the probiotic or other supplementation to metabolic improvement cannot be determined from this case, the intervention successfully motivated the patient to pursue and maintain lifestyle modifications that he had previously been unable to sustain. The probiotic recommended to this patient works by stimulating GLP-1 and ameliorating the alterations in the gut microbiome known to both precede and accompany T2D in humans. Hypothesized mechanisms of action are shown in **Figure 2**.

The probiotic formulation used by the patient was shown to significantly improve glycemic control in patients with T2D inadequately controlled with metformin alone, primarily by reducing spikes of plasma glucose following food ingestion (7). Following use for 12 weeks, a placebo-adjusted reduction in A1c (%) of -0.6 was observed. The formulation was well-tolerated, with reported adverse events that were primarily gastrointestinal in nature, occurring less frequently than in the placebo-control group.

A limitation of this case report is its focus on a single patient’s clinical practice data in a specific context, without a full evaluation of the patient’s lifestyle and overall health. Causality is ultimately unclear, since the favorable metabolic improvements observed in the patient over the 6-month period may be influenced by multiple, overlapping factors: dietary changes (fiber in particular, which was unquantified in this case), supplementation (for example, omega-3 for improving the patient’s lipid profile), exercise frequency, testosterone-driven changes in body composition (8), and weight loss.

Moreover, only one probiotic product was used, whereas several other dietary supplements, including some probiotics (9–11) with potentially different mechanisms of action, are shown to improve aspects of metabolic health in patients with a similar presentation. However, the current product is unique in using a combination of anaerobic probiotic strains independently linked with metabolic health improvements through the gut microbiome.

These results may help generate hypotheses for randomized controlled trials evaluating the efficacy of targeted probiotic supplementation in controlling metabolic parameters relevant to health as well as enhancing adherence to non-pharmacological interventions. Positive outcomes from properly designed studies addressing these issues may enhance the information in current treatment guidelines.

## 5 Patient perspective

This patient was initially hesitant to reengage in diabetes management but became motivated when presented with a non-pharmacological plan combining lifestyle and targeted support from probiotics and other supplements. With the comprehensive treatment plan, including TRT, he reported noticeable improvements in energy, libido, and overall well-being within the first few months, which reinforced continued adherence to diet and exercise measures, as well as supplement use. The patient expressed satisfaction and confidence in achieving better metabolic control and body composition changes without resuming traditional diabetes medications.

## 6 Conclusion

Overall, clinical management of T2D has fundamentally shifted with the introduction of potent, long-acting GLP-1 agonists and dual GLP-1 and GIP receptor agonists, along with multiple SGLT-2 antagonists, which improve glucose control and usually lead to clinically significant weight loss and reduced cardiovascular risk (12). However, these agents have serious side effects that occur infrequently but may be life-threatening. This report describes a patient for whom supplements, including a probiotic designed to assist in dietary management of T2D, alongside renewed attention to diet and exercise goals, was more acceptable than a pharmacological agent, and resulted in clinically relevant metabolic improvements.

Multiple factors likely contributed to the metabolic health progress observed over 6 months, including weight loss and favorable changes in body composition, consistent with improved insulin sensitivity. However, the probiotic employed was shown in a randomized clinical trial to improve aspects of metabolic health in individuals with type 2 diabetes and is a likely contributor to the clinically meaningful difference in A1c levels observed in the patient as early as 3 months.

The case demonstrates the success of a combined approach to diabetes management: lifestyle measures enhanced by supplementation that included a daily probiotic, shown to help improve metabolic parameters in individuals with T2D. A larger trial is required to evaluate the wider applicability and long-term durability of this approach.

## Data Availability

The original contributions presented in the study are included in the article/supplementary material. Further inquiries can be directed to the corresponding author.
